# Twelve-Month Real-World Outcomes of Tezepelumab in Severe Asthma: Clinical Remission, Biomarker Changes, and Trigger Burden—A SANI Multicenter Cohort

**DOI:** 10.3390/jpm16060321

**Published:** 2026-06-15

**Authors:** Stefania Nicola, Simone Negrini, Fulvia Ribolla, Giuseppe Guida, Rocco Francesco Rinaldo, Benedetta Bondi, Iuliana Badiu, Federica Corradi, Anna Quinternetto, Ilaria Vitali, Luca Lo Sardo, Benedetta Crida, Linda Mhimid, Sofia Luisa Tocci, Marcelo Teocchi, Asia Milione, Marta Marengo, Enrico Heffler, Giorgio Walter Canonica, Francesco Blasi, Pierluigi Paggiaro, Marzia Boem, Stefania Basiglio, Lucrezia Alessi, Fulvio Braido, Fabio Luigi Massimo Ricciardolo, Paolo Solidoro, Diego Bagnasco, Luisa Brussino, on behalf of the SANI Study Group

**Affiliations:** 1 Immunology and Allergy Unit, Azienda Ospedaliera Ordine Mauriziano di Torino, 10128 Turin, Italy; stefania.nicola@unito.it (S.N.); ibadiu@mauriziano.it (I.B.); fcorradi@cittadellasalute.to.it (F.C.); annaquinternetto@gmail.com (A.Q.); ilaria.vitali1@gmail.com (I.V.); llosardo@mauriziano.it (L.L.S.); linda.mhimid@unito.it (L.M.); sofialuisa.tocci@unito.it (S.L.T.); marcelo.teocchi@unito.it (M.T.); asia.milione@unito.it (A.M.); marta.marengo@unito.it (M.M.); marzia.boem@unito.it (M.B.); s.basiglio@unito.it (S.B.); luisa.brussino@unito.it (L.B.); 2 Department of Medical Sciences, University of Turin, 10126 Turin, Italy; 3 Pneumology Unit, City of Health and Science, Molinette Hospital, Department of Medical Sciences, University of Turin, 10126 Turin, Italy; fulvia.ribolla@gmail.com (F.R.); roccofrancesco.rinaldo@unito.it (R.F.R.); paolo.solidoro@unito.it (P.S.); 4 Severe Asthma, Rare Lung Disease and Respiratory Pathophysiology Unit, San Luigi Gonzaga University Hospital, Orbassano, 10043 Turin, Italy; giuseppe.guida@unito.it (G.G.); fabioluigimassimo.ricciardolo@unito.it (F.L.M.R.); 5 Allergy and Respiratory Diseases, IRCCS Policlinico San Martino, University of Genoa, 16132 Genoa, Italy; bennina.bondi@gmail.com (B.B.); fulvio.braido@unige.it (F.B.); diego.bagnasco@unige.it (D.B.); 6 Struttura Semplice Dipartimentale Pediatric Allergology Unit, Regina Margherita Children Hospital, Azienda Ospedaliero-Universitaria (AOU) Città Della Salute e Della Scienza, 10126 Turin, Italy; bcrida@cittadellasalute.to.it (B.C.); lalessi@cittadellasalute.to.it (L.A.); 7 Personalized Medicine Asthma and Allergy, IRCCS Humanitas Research Hospital, 20089 Rozzano, Italy; enrico.heffler@hunimed.eu (E.H.); giorgio_walter.canonica@hunimed.eu (G.W.C.); 8 Department of Biomedical Sciences, Humanitas University, Pieve Emanuele Milan, 20072 Milan, Italy; 9 Department of Pathophysiology and Transplantation, Università degli Studi di Milano, 20122 Milan, Italy; francesco.blasi@unimi.it; 10 Respiratory and Cystic Fibrosis Unit, Internal Medicine Department, Fondazione IRCCS Ca’ Granda Ospedale Maggiore Policlinico Milano, 20122 Milan, Italy; 11 Department of Surgery, Medicine, Molecular Biology and Critical Care, University of Pisa, 56126 Pisa, Italy; pierluigi.paggiaro@unipi.it

**Keywords:** severe asthma, tezepelumab, thymic stromal lymphopoietin, trigger burden, Asthma Trigger Inventory, clinical remission, real-world evidence, SANI, biologics, type 2 inflammation

## Abstract

**Background/Objectives**: Tezepelumab targets thymic stromal lymphopoietin and has broad efficacy in severe asthma, yet real-world evidence on patient-reported trigger burden remains limited. We assessed 12-month outcomes after tezepelumab, focusing on clinical remission, biomarkers, and trigger profiling as complementary dimensions of response. **Methods**: In this multicenter longitudinal real-world observational cohort based on routine clinical follow-up and Severe Asthma Network in Italy (SANI) registry data, 43 adults with severe asthma treated with tezepelumab at four Italian SANI reference centers were evaluated at baseline and, when available, after 1, 3, 6, and 12 months. Outcomes included exacerbations, lung function, type 2 biomarkers, the Asthma Control Test, SNOT-22, trigger categories, Asthma Trigger Inventory (ATI) scores, and SANI-defined clinical remission. **Results**: Among 22 patients with 12-month follow-up data, mean annualized exacerbations decreased from 4.30 ± 2.77 to 0.36 ± 0.49 (*p* < 0.001), and 14/22 (63.6%) were exacerbation-free. Asthma control improved, whereas FEV1 remained stable. FeNO and blood eosinophils decreased at selected time points. The number of reported trigger categories was lower at 6 months (*p* < 0.001), and physical exertion, smoke, irritants, and infection-related ATI domains improved longitudinally. Complete clinical remission was achieved in 5/22 patients (22.7%). **Conclusions**: Tezepelumab was associated with reduced exacerbations, improved asthma control, and lower patient-reported trigger burden. Structured trigger profiling may provide an exploratory patient-centered dimension for assessing treatment response in severe asthma.

## 1. Introduction

Asthma remains a major global health challenge, with approximately 260 million prevalent cases and more than 430,000 deaths worldwide in 2021. Although severe asthma affects only a minority of patients, it accounts for a disproportionate share of morbidity, healthcare utilization, treatment burden, and mortality [1,2]. Exacerbations are a major driver of this burden, contributing to emergency department visits, hospitalizations, systemic corticosteroid exposure, impaired quality of life, productivity loss, and potentially accelerated lung function decline [3,4].

A major challenge in severe asthma is its marked biological heterogeneity. Phenotype and endotype are related but distinct constructs, yet robust biomarkers capable of defining them precisely at the individual-patient level remain limited in routine practice. Blood eosinophils, fractional exhaled nitric oxide, total IgE, allergen sensitization, and sputum cytology can all be informative, but they are variably influenced by corticosteroid exposure, comorbidities, environmental factors, and temporal variability. Thus, currently available biomarkers should be regarded as useful but incomplete surrogates of underlying disease biology [2,5,6].

This limitation is particularly relevant because asthma worsening is often precipitated by exposure to triggers acting at the host–environment interface. Increasing evidence supports an important role of the airway epithelium in asthma pathobiology. By sensing viruses, allergens, pollutants, cigarette smoke, irritants, and physical stress, the airway epithelium can initiate alarmin release and downstream inflammatory responses associated with airway hyperresponsiveness and exacerbation susceptibility [7,8]. In this context, patient-reported triggers may be viewed as clinically meaningful correlates of disease vulnerability. Notably, in the CHRONICLE cohort, a greater number of patient-reported triggers was associated with poorer asthma control, lower quality of life, and greater work impairment [9].

Among epithelial alarmins, thymic stromal lymphopoietin (TSLP) is of particular interest because it links epithelial stress to multiple downstream inflammatory pathways and has been associated with airway hyperresponsiveness independently of eosinophilic inflammation [7,8]. This rationale underpins tezepelumab, a monoclonal antibody targeting TSLP. Therefore, tezepelumab has been shown in randomized clinical trials to significantly reduce annualized asthma exacerbation rates in severe uncontrolled asthma [10,11,12,13,14].

However, available tezepelumab literature has focused mainly on exacerbations, symptom control, lung function, and biomarkers, whereas evidence regarding patient-perceived trigger burden remains limited and largely indirect [15,16,17]. Against this background, we aimed to evaluate the real-world effectiveness of tezepelumab in severe asthma, not only in terms of exacerbation reduction and asthma control, but also with respect to longitudinal changes in trigger burden. The primary objective of this study was to describe longitudinal changes in exacerbation burden and asthma control during tezepelumab treatment in routine clinical practice. A secondary objective was to explore changes in patient-reported trigger burden, including the number of trigger categories and selected ATI domain scores, as an exploratory dimension of treatment response [7,9,15,16,17].

## 2. Materials and Methods

### 2.1. Study Design and Population

We conducted a multicenter longitudinal real-world observational cohort study of adult patients with severe asthma who initiated tezepelumab at four university hospitals in northern Italy, all of which are Severe Asthma Network in Italy (SANI) reference centers, between February 2024 and December 2025: the Allergy and Immunology Unit of Ordine Mauriziano Hospital, Turin, Italy; the Pneumology Unit and the Pediatric Pulmonology Unit of AOU Città della Salute e della Scienza di Torino [respectively Molinette Hospital and Regina Margherita Children Hospital], Turin, Italy; the Severe Asthma, Rare Lung Disease and Respiratory Pathophysiology Unit of AOU San Luigi, Orbassano, Turin, Italy; and the Allergy and Respiratory Diseases Unit of Policlinico San Martino Hospital, Genoa, Italy.

Patients were identified from site clinical databases and routine specialist follow-up records. Eligible patients were adults with severe asthma defined according to GINA criteria as asthma remaining uncontrolled despite confirmed adherence to maximal optimized high-dose ICS-LABA treatment, correct inhaler technique, and management of contributory factors, or worsening when high-dose treatment was reduced [18,19]. Additional eligibility criteria were initiation of tezepelumab during the study period and availability of baseline data. Because this was a real-world observational study based on routine care, follow-up assessments were not uniformly complete across variables and visits.

Baseline demographic and clinical variables included age, sex, smoking status, body mass index (BMI), asthma phenotype, allergic status, disease duration, annual exacerbation burden before treatment initiation, previous biologic exposure, and comorbidities. Comorbidities were extracted from site clinical databases, specialist follow-up records, and available medical documentation, rather than being based solely on patient self-report. T2-related comorbidities, including nasal polyposis, allergic rhinitis, and nonsteroidal anti-inflammatory drug intolerance, were also recorded.

The study was approved by the local ethics committee of the participating centers [SANI Registry—“Area Vasta Nord-Ovest Toscana,” Ethics Committee protocol number 73714, study number 1245/2016, approved on 7 December 2016]. All patients were enrolled in the SANI registry for severe asthma, and written informed consent was obtained from all participants before data collection.

### 2.2. Data Collection and Study Variables

Patients were evaluated at baseline and, whenever data were available, after 1, 3, 6, and 12 months of treatment as part of routine specialist follow-up. Longitudinal assessments included lung function, inflammatory biomarkers, patient-reported outcomes, and trigger-related measures. Because this was a real-world observational cohort rather than a protocol-mandated prospective trial, follow-up visits and outcome availability were not uniform across patients and time points.

Lung function assessment included forced expiratory volume in 1 s (FEV1, L). Type 2 inflammatory biomarkers included fractional exhaled nitric oxide (FeNO), total immunoglobulin E (IgE), and blood eosinophil count. Clinical and sinonasal outcomes included the Asthma Control Test (ACT) and the 22-item Sino-Nasal Outcome Test (SNOT-22).

Asthma trigger burden was assessed in two complementary ways. First, the number and type of reported trigger categories were recorded at each visit using the same predefined checklist during routine clinical assessment; recorded categories included allergens, physical exertion, temperature change, smoke, laughter, and infection. Trigger-category data were collected at baseline and planned for collection at 3, 6, and 12 months, although actual availability varied by visit. Second, trigger burden was further explored using selected domains adapted from the original Asthma Trigger Inventory (ATI), including psychological triggers, physical exertion, smoke, irritants, and infections, administered in Italian for clinical use. The psychological trigger domain referred to patient-perceived asthma worsening related to emotional stress or psychological conditions; the physical exertion domain referred to exercise- or activity-related symptom worsening; the smoke domain referred to perceived asthma worsening related to tobacco smoke exposure, including active or passive cigarette smoke exposure when reported; the irritants domain included non-tobacco irritant exposures, such as strong odors, fumes, chemical irritants, cleaning products, perfumes, and air pollution or other environmental irritants reported during routine clinical assessment; and the infections domain referred to respiratory infections perceived as asthma triggers. These domains reflected patient-reported perceived triggers and were not intended to quantify objective exposure levels or to distinguish specific exposure sources such as wood smoke or burn pit exposure. ATI domain scores were analyzed longitudinally across baseline and 1, 3, 6, and 12 months, whenever available [20].

### 2.3. Definitions

Allergic asthma is asthma associated with sensitization to aeroallergens that leads to asthma symptoms and airway inflammation upon exposure [21].

The annualized exacerbation rate before tezepelumab initiation referred to the number of asthma exacerbations recorded during the 12 months preceding treatment initiation.

Exacerbations were defined as clinically relevant episodes of asthma worsening requiring systemic corticosteroids and/or asthma-related urgent healthcare utilization, including emergency department visit or hospitalization, according to routine specialist clinical documentation.

Clinical remission at 12 months was assessed according to the SANI framework [22]. Briefly, complete remission was defined by the concomitant presence of all of the following at 12 months: absence of maintenance oral corticosteroid use, absence of exacerbations, adequate symptom control defined as ACT score ≥ 20, and stable lung function. Partial remission was defined as fulfillment of all remission criteria with the exception of one of the four components.

Because of the real-world prospective nature of the dataset, stable lung function was defined as absence of clinically meaningful deterioration in follow-up FEV1 relative to baseline. For the purposes of this study, stable lung function was defined as no decline in FEV1 greater than 15% from baseline at 12 months, consistent with MCID-based approaches proposed for longer-term asthma assessment [21].

### 2.4. Outcomes

The main outcomes of interest were longitudinal changes in exacerbation burden and asthma control, assessed by the annualized exacerbation rate and Asthma Control Test (ACT), during tezepelumab treatment in routine clinical practice.

Additional exploratory outcomes included FEV1, FeNO, total IgE, blood eosinophil count, SNOT-22, number and prevalence of trigger categories, Asthma Trigger Inventory (ATI) domain scores, and 12-month clinical remission. A specific exploratory objective of the study was to assess whether tezepelumab treatment was associated with a reduction in patient-reported trigger burden, both in terms of the number of trigger categories and the intensity of selected ATI trigger domains.

### 2.5. Statistical Analysis

Statistical analyses were conducted using Python 3.11 with the latest stable versions of pandas, scipy, and statsmodels available at the time of analysis, and cross-validated with Stata/SE 18.0 (StataCorp LLC, College Station, TX, USA) for consistency.

Continuous variables are summarized according to distribution as mean ± standard deviation (SD) or median with interquartile range (IQR), as appropriate. Categorical variables are reported as counts and percentages.

Given the observational design and uneven completeness of repeated assessments, longitudinal analyses were primarily based on available-case data at each time point. For FEV1, FeNO, total IgE, blood eosinophil count, ACT, SNOT-22, and number of trigger categories, baseline-versus-follow-up comparisons were performed using paired tests restricted to subjects with data available at both relevant time points. Because several variables showed a non-Gaussian distribution and sample size differed across visits, paired Wilcoxon signed-rank tests were used for these comparisons.

To complement these pairwise analyses, global within-subject changes across repeated visits were assessed using the Friedman test on complete cases only. Accordingly, repeated-measures global tests and baseline-versus-time-point paired comparisons should be interpreted as complementary analyses performed under different missing-data constraints.

Because prevalence of individual trigger categories was analyzed as a repeated binary outcome, Cochran’s Q test was used across baseline, 3 months, and 6 months. Twelve-month trigger prevalence was considered descriptive only due to limited data availability.

ATI domain scores were treated as approximately continuous for analysis. Global changes over time were assessed using repeated-measures analysis of variance (ANOVA) on complete cases, and pairwise comparisons between baseline and each follow-up visit were performed using paired *t*-tests. Holm correction was applied to adjust for multiple pairwise comparisons within each ATI domain.

All statistical tests were two-sided, and *p* < 0.05 was considered statistically significant. Because of the exploratory nature of several longitudinal comparisons, adjustment for multiple testing was limited to within-domain ATI pairwise analyses. Accordingly, *p* values outside the prespecified Holm-adjusted ATI pairwise comparisons are reported as nominal and should be interpreted cautiously. No imputation for missing data was performed. Because follow-up completeness varied across visits and outcomes, complete-case analyses may reflect a subset of participants and were interpreted accordingly.

## 3. Results

### 3.1. Demographic Data and Comorbidities

The study sample included 43 patients, with a mean age of 57.4 ± 15.1 years; 34 patients (79.1%) were female. Regarding smoking status, 23 patients (53.5%) were never smokers, 15 (34.9%) were former smokers, and 5 (11.6%) were current smokers. Mean body mass index (BMI) was 27.4 ± 5.9 kg/m^2^; 34.2% of patients were normal weight, whereas obesity was recorded in 3 of 43 patients (7.0%) (Table 1).

Overall, 36 patients (83.7%) had at least one recorded general comorbidity. The distribution of the main comorbidities is reported in Table 2. Gastroesophageal reflux disease (GERD) was the most frequent comorbidity, affecting 16 patients (37.2%), followed by obstructive sleep apnea syndrome (OSAS) in 9 (20.9%). Osteoporosis/osteopenia and autoimmune thyroid disease/hypothyroidism were each reported in 6 patients (14.0%), while hypertension was present in 5 (11.6%).

### 3.2. Asthma Phenotypes and Therapies

Overall, this was a heavily treated and predominantly biologic-experienced severe asthma cohort, with 69.8% of patients previously exposed to at least one biologic agent and 72.1% receiving maintenance oral corticosteroids at baseline. A T2-high asthma phenotype was reported in 34 of 43 patients (79.1%), whereas 9 patients (20.9%) were classified as having non-T2 asthma. Allergic asthma was present in 26 patients (60.5%). Median disease duration was 23.5 years [IQR 10.0–43.0]. T2-related comorbidities were common, with allergic rhinitis reported in 26 patients (60.5%), nasal polyposis in 14 (32.6%), and NSAID intolerance in 7 (16.3%). Overall, 33 patients (76.7%) had at least one recorded T2-related comorbidity (Table 3).

Of the 43 patients included in the baseline cohort, 30 (69.8%) had received at least one biologic therapy before starting tezepelumab, whereas 13 (30.2%) had no previously recorded biologic treatment. Among prior biologic exposures, omalizumab was the most frequent (16 patients), followed by dupilumab (15 patients), mepolizumab (8 patients), and benralizumab (6 patients). Because some patients had received more than one biologic agent before tezepelumab initiation, these categories were not mutually exclusive. In addition, 1 of 43 patients (2.3%) had previously received allergen immunotherapy (AIT), specifically for grass pollen, whereas the remaining 42 patients (97.7%) had no AIT recorded.

At baseline, all 43 patients (100.0%) were receiving combination therapy with ICS-LABA, while 37 of 43 patients (86.0%) were also receiving LAMA as part of triple inhaled therapy.

Maintenance OCS use was documented in 31 of 43 patients (72.1%), with a mean prednisone dose of 10.2 ± 6.5 mg/day and a median dose of 10 mg/day (IQR, 5–12.5 mg/day). Following tezepelumab initiation, OCS exposure progressively decreased. At 1 month, 8 of 24 patients with available treatment data (33.3%) remained on maintenance OCS, with a mean prednisone dose of 10.3 ± 9.3 mg/day. At 3 months, 10 of 43 patients (23.3%) were still receiving OCS, with a mean dose of 8.0 ± 7.9 mg/day. At 6 months, only 3 of 43 patients (7.0%) remained on maintenance OCS, and by 12 months, this number had fallen to 2 of 22 patients (9.1%).

### 3.3. Exacerbation Burden Before and After Tezepelumab

Before tezepelumab initiation, a total of 156 exacerbations were recorded in the overall cohort, corresponding to a mean of 3.63 ± 2.28 exacerbations per patient-year. The most commonly reported causes of exacerbation during the 12 months preceding tezepelumab initiation are summarized in Table 4. Briefly, infections were the most frequently reported cause, affecting 20 patients (46.5%), followed by physical exertion in 16 (37.2%), allergens in 12 (27.9%), and smoking in 11 (25.6%). Strong odors/irritants were rarely reported, being identified in only 1 patient (2.3%).

Among the 22 patients with available 12-month follow-up data, mean exacerbations/year decreased from 4.30 ± 2.77 at baseline to 0.36 ± 0.49 at 12 months, corresponding to an absolute reduction of 3.93 exacerbations/year and a relative reduction of 91.5%. This reduction was statistically significant on paired analysis (Wilcoxon signed-rank test, *p* < 0.001). Consistently, the median exacerbation rate decreased from 4.0 [IQR 2.25–5.75] at baseline to 0.0 [IQR 0.0–1.0] at 12 months. Overall, 14 of 22 patients (63.6%) were free from exacerbations at 12 months. 

**Figure 1:**
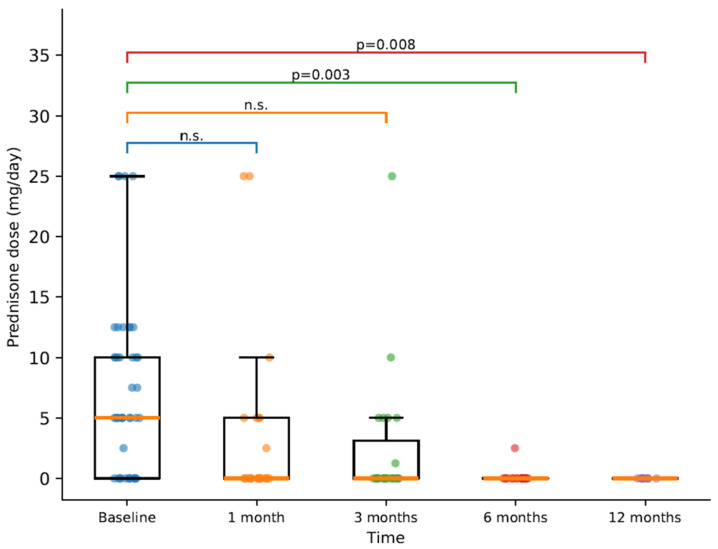
Maintenance oral corticosteroid dose over time in the enrolled cohort. Dots represent individual patient values at each time point. Colors are used only to visually distinguish the different time points and do not encode any additional variable. Box-and-whisker plots summarize the dis-tribution of prednisone dose at each time point; the orange horizontal line indicates the median. Brackets indicate paired comparisons, with the corresponding *p* values shown above the brackets. n.s., not significant.

The longitudinal change in maintenance OCS dose is shown in Figure 1.

### 3.4. Longitudinal Analysis of Clinical, Inflammatory, and Trigger-Related Variables

Longitudinal comparisons were performed between baseline, 1 month, 3 months, 6 months, and 12 months whenever data were available.

#### 3.4.1. Lung Function

Mean FEV1 was 2.18 ± 0.95 L at baseline, 2.21 ± 1.03 L at 1 month, 2.23 ± 0.97 L at 3 months, 2.27 ± 1.05 L at 6 months, and 2.06 ± 1.25 L at 12 months. No significant paired baseline-versus-follow-up differences were observed, and the overall repeated-measures comparison was not significant (overall *p* = 0.866). FEV1 did not show statistically significant within-subject change in the available paired analyses, although the small complete-case sample precludes any inference of equivalence or absence of effect (Table S1).

#### 3.4.2. Type 2 Inflammatory Biomarkers

FeNO showed lower values during early follow-up. Median FeNO was 20.0 ppb [IQR 12.2–34.8] at baseline, 18.0 [IQR 12.0–32.0] at 1 month, 18.0 [IQR 11.0–31.0] at 3 months, 15.0 [IQR 12.0–29.0] at 6 months, and 21.0 [IQR 9.8–33.0] at 12 months. Compared with baseline, paired reductions were significant at 1 month (*p* = 0.004), 3 months (*p* = 0.014), and 6 months (*p* = 0.040). The overall repeated-measures comparison was not significant (overall *p* = 0.525). Given the limited number of observations at 12 months, the 12-month FeNO value should be interpreted descriptively.

Total IgE values were unevenly collected at follow-up. Median total IgE was 89.0 kU/L [IQR 35.0–248.0] at baseline, 586.5 [57.2–1193.5] at 1 month, 112.0 [31.0–248.0] at 3 months, 211.0 [37.9–303.0] at 6 months, and 29.8 [17.1–129.3] at 12 months. Because follow-up availability was very limited and no global longitudinal test was available, IgE results were considered descriptive.

Blood eosinophil count decreased at selected time points. Median blood eosinophil count was 140.0 cells/µL [IQR 100.0–300.0] at baseline, 85.0 [37.5–130.0] at 1 month, 140.0 [85.0–285.0] at 3 months, 115.0 [90.0–187.5] at 6 months, and 100.0 [30.0–170.0] at 12 months. Compared with baseline, paired reductions were significant at 1 month (*p* < 0.001) and 6 months (*p* = 0.016). The overall repeated-measures comparison was not significant (overall *p* = 0.279). Given the limited number of observations at 12 months, the 12-month eosinophil value should be interpreted descriptively (Table S1).

**Figure 2:**
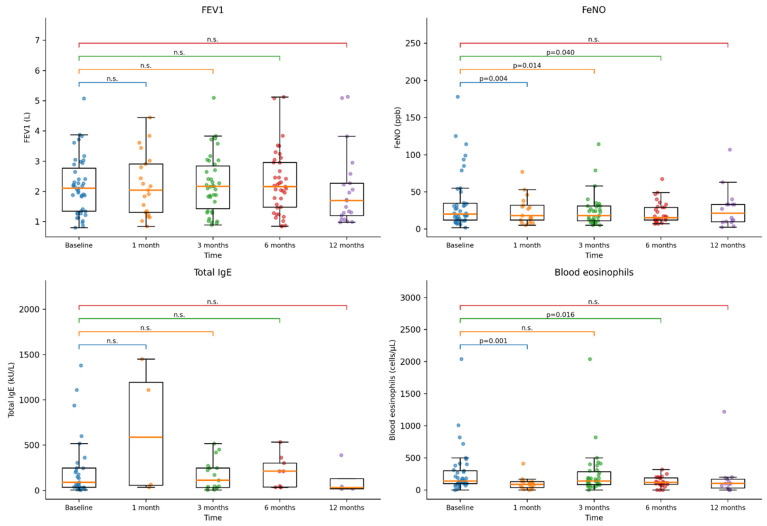
Longitudinal changes in lung function and inflammatory biomarkers over time. Colored dots represent individual patient values at each time point. Colors are used only to visually dis-tinguish the different time points and do not encode any additional variable. Box-and-whisker plots summarize the distribution of each variable at each time point; the orange horizontal line indicates the median. Brackets indicate paired comparisons between baseline and the corresponding fol-low-up time points, with *p* values shown above the brackets. n.s., not significant. FEV1, forced ex-piratory volume in 1 s; FeNO, fractional exhaled nitric oxide; IgE, immunoglobulin E.

The longitudinal changes in FEV1 and inflammatory biomarkers are shown in Figure 2.

#### 3.4.3. Clinical Outcomes and Symptom Scores

ACT scores were significantly higher over follow-up than at baseline (Table S1). Mean ACT was 14.86 ± 4.85 at baseline, 17.83 ± 5.48 at 1 month, 16.34 ± 6.20 at 3 months, 18.39 ± 5.43 at 6 months, and 18.29 ± 4.69 at 12 months. Paired comparisons versus baseline were significant at 1 month (*p* = 0.024), 3 months (*p* = 0.019), 6 months (*p* = 0.004), and 12 months (*p* = 0.002). The overall repeated-measures comparison was also significant (*p* = 0.014).

**Figure 3:**
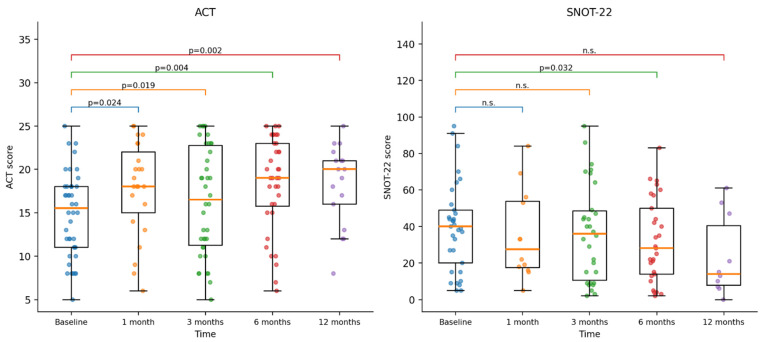
Longitudinal changes in patient-reported outcomes over time. Colored dots represent individual patient values at each time point. Colors are used only to visually distinguish the different time points and do not encode any additional variable. Box-and-whisker plots summarize the dis-tribution of each variable at each time point; the orange horizontal line indicates the median. Brackets indicate paired comparisons between baseline and the corresponding follow-up time points, with *p* values shown above the brackets. n.s., not significant. ACT, Asthma Control Test; SNOT-22, 22-item Sino-Nasal Outcome Test.

SNOT-22 values were lower over follow-up than at baseline. Median SNOT-22 was 40.0 [IQR 20.0–49.0] at baseline, 27.5 [17.5–53.8] at 1 month, 36.0 [10.5–48.5] at 3 months, 28.0 [14.0–50.0] at 6 months, and 14.0 [7.8–40.5] at 12 months. A paired reduction versus baseline was reported at 6 months (*p* = 0.032). No overall repeated-measures *p* value was available for SNOT-22, and 12-month values should be interpreted descriptively because of sparse follow-up data. Figure 3 and Table S1 display the aforementioned data.

#### 3.4.4. Number of Triggers and Trigger Profile

The number of reported trigger categories was assessed at baseline, 3 months, and 6 months; 12-month data were not included in inferential analysis because of limited availability (Table S2). Mean number of triggers was 3.79 ± 1.10 at baseline, 3.88 ± 1.29 at 3 months, and 3.05 ± 1.72 at 6 months. In paired available-case analyses, the number of reported trigger categories was lower at 6 months than at baseline (*p* < 0.001). No overall repeated-measures *p* value was available for this variable.

At baseline, the most frequently reported trigger categories were physical exertion (38/43, 88.4%), infection (36/43, 83.7%), and smoke (30/43, 69.8%), followed by allergens (23/43, 53.5%), laughter (20/43, 46.5%), and temperature change (17/43, 39.5%). At 3 months, trigger prevalence was as follows: physical exertion, 42/43 (97.7%); infection, 40/43 (93.0%); smoke, 33/43 (76.7%); allergens, 27/43 (62.8%); laughter, 16/43 (37.2%); and temperature change, 9/43 (20.9%). At 6 months, trigger prevalence was as follows: physical exertion, 34/41 (82.9%); infection, 25/41 (61.0%); smoke, 22/41 (53.7%); allergens, 16/41 (39.0%); laughter, 16/41 (39.0%); and temperature change, 12/41 (29.3%). Descriptive prevalences are reported using all available observations at each visit. Inferential comparisons using Cochran’s Q were performed only in subjects with complete data across baseline, month 3, and month 6, as detailed in Supplementary Table S2.

Across baseline, 3 months, and 6 months, the prevalence of several individual trigger categories changed significantly over time, including allergens (*p* = 0.009), physical exertion (*p* = 0.009), temperature change (*p* = 0.002), smoke (*p* = 0.008), and infection (*p* < 0.001), whereas laughter did not show a significant change (*p* = 0.074). Twelve-month trigger prevalence was considered descriptive only because of sparse and variable data availability across categories.

#### 3.4.5. ATI Trigger Scores

ATI domain scores were analyzed longitudinally across baseline and 1, 3, 6, and 12 months.

Repeated-measures ANOVA for ATI domains was performed on complete cases only; the complete-case *n* for each domain is reported in Supplementary Table S3.

Psychological trigger scores did not show significant longitudinal change (overall *p* = 0.431). Physical exertion scores changed significantly over time (overall *p* = 0.012), with a significant Holm-corrected paired comparison at 6 months (Holm *p* = 0.011). Smoke-related scores also changed significantly (overall *p* < 0.001), with a significant reduction at 6 months (Holm *p* = 0.009). Irritant-related scores showed significant longitudinal changes (overall *p* < 0.001), with significant Holm-corrected reductions at 3 months (Holm *p* = 0.004), 6 months (Holm *p* < 0.001), and 12 months (Holm *p* = 0.007). Infection-related scores also changed significantly over time (overall *p* < 0.001), with significant Holm-corrected reductions at 3 months (Holm *p* = 0.025), 6 months (Holm *p* < 0.001), and 12 months (Holm *p* = 0.010). These findings should be interpreted in light of the limited complete-case sample sizes reported in the supplementary analyses. These findings should be interpreted in light of the limited complete-case sample sizes reported in the supplementary analyses.

**Figure 4:**
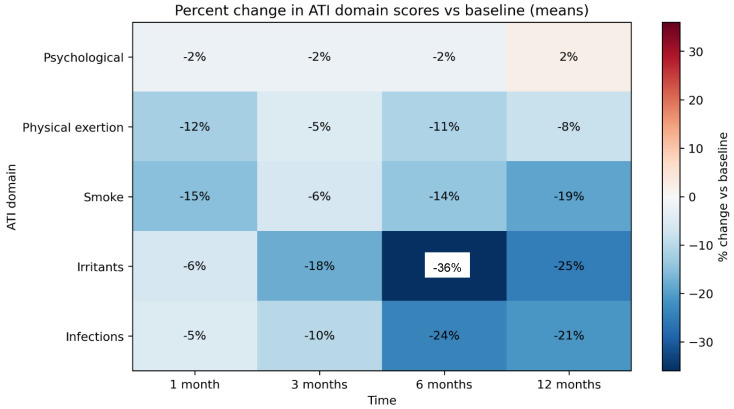
Percentage change in ATI domain score vs. baseline.

These percentage changes in ATI domain scores versus baseline are shown in Figure 4.

### 3.5. Asthma Remission

Among the 22 patients with available 12-month follow-up, 5 (22.7%) fulfilled all prespecified SANI-based criteria for complete clinical remission at 12 months. This corresponded to 11.6% of the overall study population. Using the same framework, 10 of 22 patients (45.5%) achieved at least partial remission.

## 4. Discussion

In this multicenter real-world cohort of patients with severe asthma, treatment with tezepelumab was associated with improved asthma control, reductions in selected type 2 inflammatory biomarkers at specific time points, improvement in sinonasal symptom burden, and lower patient-reported trigger burden over follow-up, whereas lung function remained overall stable. Taken together, these findings suggest that, in this cohort and within the limits of outcome availability, changes in symptom control, exacerbation burden, selected inflammatory indices, and patient-reported trigger burden were more readily detectable than changes in spirometry [23,24]. This overall pattern is broadly consistent with evidence from the tezepelumab clinical development program and emerging real-world experience [10,11,12,13,14].

The improvement in ACT observed in our cohort is clinically relevant, particularly because symptom control is a key treatment target in severe asthma and may influence treatment persistence and patient-perceived benefit. By contrast, FEV1 remained overall unchanged. This pattern is not unexpected in severe asthma populations with long-standing disease, in whom clinically meaningful improvement may occur without major changes in absolute spirometric measures [11,12,13,14]. The biomarker findings were also directionally consistent with the expected effects of upstream TSLP blockade, with significant reductions in FeNO during early follow-up and in blood eosinophil count at selected time points, although these changes were not uniform across all visits and 12-month data were limited for some biomarkers. Similarly, SNOT-22 values were lower over follow-up, with a significant paired reduction reported at 6 months, suggesting potential benefit on upper-airway symptom burden, although sinonasal outcomes should be interpreted cautiously because of incomplete data, particularly at later visits [15].

A distinctive aspect of the present study is the longitudinal assessment of trigger burden. In addition to the reduction in exacerbation frequency, the number of reported trigger categories was lower at 6 months, and several ATI domains—particularly infections, smoke, irritants, and physical exertion—showed significant longitudinal improvement. These observations suggest that patient-reported trigger burden may represent an additional dimension of treatment response in severe asthma [9]. At the same time, these findings should be interpreted with appropriate caution, as the trigger analyses were based on incomplete follow-up data and some domain-specific analyses relied on small complete-case subsets.

From a clinical perspective, trigger-related outcomes may be relevant because they reflect how patients experience disease instability in everyday life. Exposure to infections, exertion, smoke, irritants, and other environmental factors often shapes daily behavior and perceived disease burden in ways that are not fully captured by isolated biomarker measurements or spirometry [2,7,9]. In this context, the observed reduction in trigger burden may reflect lower patient-reported susceptibility to symptom-provoking exposures during follow-up; however, no mechanistic inference can be drawn from this uncontrolled observational dataset [7,8,16,17].

The findings related to infection-associated triggers deserve particular interest and interpretation. Infections were among the most frequently reported causes of exacerbation at baseline and one of the ATI domains showing longitudinal improvement. This observation may be clinically relevant, given the known role of respiratory infections in asthma worsening [7,8,16]. However, this should not be interpreted as evidence of a direct anti-infective effect of tezepelumab, but rather as suggesting that treatment may be associated with a lower likelihood that infectious exposures are perceived or experienced as triggers of clinically relevant asthma worsening, possibly as a result of upstream TSLP blockade and its effects on the airway epithelium. Similar considerations apply to smoke- and irritant-related domains.

The interpretation of exertional triggers also warrants nuance. Physical exertion remained a frequently reported trigger category across follow-up, yet ATI physical exertion scores improved significantly over time. This pattern may suggest not the disappearance of exertion as a provoking context, but a possible reduction in the intensity or clinical consequences of exertion-related worsening. Although speculative, such a pattern would be clinically relevant, as it may reflect improved day-to-day tolerance to activity even in the absence of marked spirometric change [9,12].

Taken together, these observations support the view that trigger profiling may provide complementary clinical information alongside conventional efficacy outcomes such as exacerbations, symptom control, and biomarkers [2,7,9]. This may be of interest particularly for therapies such as tezepelumab, whose upstream target is biologically linked to airway responses to a broad range of environmental and host-related stimuli [7,8]. Nevertheless, the present data should be considered exploratory in this regard, and no inference can be made about whether changes in trigger burden identify a specific mechanistic response pattern or predict long-term outcomes.

Several limitations should be acknowledged. This was a real-world observational cohort based on routine clinical follow-up and registry-derived data rather than protocol-mandated visits. Accordingly, the study had a relatively small sample size, incomplete follow-up data for several variables, and no comparator group. Missing data across time points therefore reflect real-world data availability and may introduce selection bias in longitudinal available-case analyses. In addition, as expected in a real-world study, the cohort was predominantly characterized by T2-high disease and a substantial burden of upper-airway comorbidity, which may limit generalizability to other severe asthma populations. These limitations are particularly relevant when interpreting trigger-related findings, which, although clinically interesting, require confirmation in larger prospective studies with more complete longitudinal assessment. Because smoking status may influence smoke- and irritant-related trigger perception, this issue also deserves consideration. In the present cohort, smoking status was recorded at baseline, but smoke- and irritant-related triggers were collected as patient-reported perceived triggers and were not prospectively structured to distinguish active smoking, passive smoke exposure, or other environmental irritants in sufficient detail for robust subgroup analyses. Moreover, the small number of current smokers limited the reliability of stratified comparisons. Therefore, the possible influence of smoking status on trigger burden and its longitudinal change should be addressed in larger prospective studies with a more detailed exposure assessment.

In conclusion, our real-world findings are consistent with clinical benefit during tezepelumab follow-up in severe asthma, particularly with respect to exacerbation burden and asthma control [10,11,12,13,14]. The observed changes in patient-reported trigger burden suggest that trigger profiling may capture aspects of treatment response not fully reflected by conventional endpoints alone [9,15,16,17]; however, these findings should be considered exploratory and hypothesis-generating and warrant confirmation in prospective studies specifically designed to evaluate the relationship between anti-TSLP therapy, trigger burden, and longer-term clinical outcomes.

## Figures and Tables

**Table 1:** Demographic and clinical characteristics of the sample.

Variable	Value
Number of patients	43
Age, years, mean ± SD	57.4 ± 15.1
Female sex, *n* (%)	34 (79.1%)
Smoking status	Never smokers: 23 (53.5%); Former smokers: 15 (34.9%); Current smokers: 5 (11.6%)
BMI, kg/m^2^, mean ± SD	27.4 ± 5.9

**Table 2:** General comorbidities.

Comorbidity	*n* (%)
GERD	16 (37.2%)
OSAS	9 (20.9%)
Osteoporosis/osteopenia	6 (14.0%)
Autoimmune thyroid disease/hypothyroidism	6 (14.0%)
Hypertension	5 (11.6%)
Nasal polyposis/rhinosinusitis	5 (11.6%)
Psoriatic disease	4 (9.3%)
Obesity	3 (7.0%)
Fibromyalgia	3 (7.0%)
Dyslipidemia	3 (7.0%)
Bronchiectasis	3 (7.0%)

**Table 3:** Asthma phenotype, allergic asthma, and T2 comorbidities.

Variable	*n* (%)
T2-high asthma phenotype	34 (79.1%)
Non-T2 phenotype	9 (20.9%)
Allergic asthma	26 (60.5%)
Nasal polyposis	14 (32.6%)
Allergic rhinitis	26 (60.5%)
NSAID intolerance	7 (16.3%)
At least one coded T2 comorbidity	33 (76.7%)

**Table 4:** Reported causes of exacerbations during the 12 months preceding tezepelumab initiation.

Reported Cause	Patients with Cause, *n* (%)
Infections	20 (46.5%)
Physical exertion	16 (37.2%)
Allergens	12 (27.9%)
Smoking	11 (25.6%)
Strong odors/irritants	1 (2.3%)

## Data Availability

The data presented in this study are available on reasonable request from the corresponding author. The data are not publicly available due to privacy and ethical restrictions.

## Supplementary Materials

The following supporting information can be downloaded at: https://www.mdpi.com/article/10.3390/jpm16060321/s1, Table S1: Longitudinal changes in biomarkers and patient-reported outcomes; Table S2: Prevalence of individual trigger categories over time; Table S3: Longitudinal changes in trigger burden and ATI domain scores.
