# Supplementary material for: Twelve-Month Real-World Outcomes of Tezepelumab in Severe Asthma: Clinical Remission, Biomarker Changes, and Trigger Burden—A SANI Multicenter Cohort
Source: J Pers Med. 2026 Jun 15;16(6):321. doi: 10.3390/jpm16060321 (PMC13301168; doi:10.3390/jpm16060321)
Supplement: Supplementary file 1 [file jpm-16-00321-s001.zip › jpm-4344112-supplementary.pdf]

Supplementary tables

Table S1. Longitudinal changes in biomarkers and patient-reported outcomes. Key paired comparisons are reported versus baseline.

Variable	Baseline	1 month	3 months	6 months	12 months	Overall p	Key paired comparisons
FEV1 (L)	2.18 ± 0.95 (n=43)	2.21 ± 1.03 (n=21)	2.23 ± 0.97 (n=38)	2.27 ± 1.05 (n=39)	2.06 ± 1.25 (n=21)	0.866	None
FeNO (ppb)	20.0 [12.2-34.8] (n=42)	18.0 [12.0-32.0] (n=17)	18.0 [11.0-31.0] (n=33)	15.0 [12.0-29.0] (n=31)	21.0 [9.8-33.0] (n=16)	0.525	1 month: p=0.004; 3 months: p=0.014; 6 months: p=0.040
Total IgE (kU/L)	89.0 [35.0-248.0] (n=29)	586.5 [57.2-1193.5] (n=4)	112.0 [31.0-248.0] (n=17)	211.0 [37.9-303.0] (n=9)	29.8 [17.1-129.3] (n=4)	NA	None
Blood eosinophils (cells/uL)	140.0 [100.0-300.0] (n=41)	85.0 [37.5-130.0] (n=16)	140.0 [85.0-285.0] (n=35)	115.0 [90.0-187.5] (n=26)	100.0 [30.0-170.0] (n=13)	0.279	1 month: p<0.001; 6 months: p=0.016
ACT	14.86 ± 4.85 (n=42)	17.83 ± 5.48 (n=23)	16.34 ± 6.20 (n=38)	18.39 ± 5.43 (n=36)	18.29 ± 4.69 (n=17)	0.014	1 month: p=0.024; 3 months: p=0.019; 6 months: p=0.004; 12 months: p=0.002
SNOT-22	40.0 [20.0-49.0] (n=33)	27.5 [17.5-53.8] (n=12)	36.0 [10.5-48.5] (n=30)	28.0 [14.0-50.0] (n=29)	14.0 [7.8-40.5] (n=10)	NA	6 months: p=0.032

Abbreviations: ACT, Asthma Control Test; FeNO, fractional exhaled nitric oxide; FEV1, forced expiratory volume in 1 second; IgE, immunoglobulin E; SNOT-22, 22-item Sino-Nasal Outcome Test.

Table S2. Prevalence of individual trigger categories over time. Data are shown as n/N (%). Overall p values derive from Cochran's Q test across baseline, 3 months, and 6 months; 12-month values are descriptive only because of sparse availability.

Trigger	Baseline	3 months	6 months	12 months	Overall p
Allergens	23/43 (53.5%)	27/43 (62.8%)	16/41 (39.0%)	12/18 (66.7%)	0.009
Physical exertion	38/43 (88.4%)	42/43 (97.7%)	34/41 (82.9%)	7/7 (100.0%)	0.009
Temperature change	17/43 (39.5%)	9/43 (20.9%)	12/41 (29.3%)	2/7 (28.6%)	0.002
Smoke	30/43 (69.8%)	33/43 (76.7%)	22/41 (53.7%)	7/7 (100.0%)	0.008
Laughter	20/43 (46.5%)	16/43 (37.2%)	16/41 (39.0%)	4/7 (57.1%)	0.074
Infection	36/43 (83.7%)	40/43 (93.0%)	25/41 (61.0%)	7/7 (100.0%)	<0.001

Table S3. Longitudinal changes in trigger burden and ATI domain scores. Continuous variables are reported as mean ± SD or median [IQR], as appropriate. Key paired comparisons are reported versus baseline.

Variable	Baseline	1 month	3 months	6 months	12 months	Overall p	Key paired comparisons
Number of triggers	3.79 ± 1.10 (n=43)	NA	3.88 ± 1.29 (n=43)	3.05 ± 1.72 (n=41)	NA	NA	6 months: p<0.001
ATI psychological triggers	1.0 [1.0-3.0] (n=32)	1.0 [1.0-2.0] (n=13)	1.0 [1.0-2.2] (n=32)	1.0 [1.0-2.0] (n=29)	1.0 [1.0-3.0] (n=13)	0.406	None
ATI physical exertion	3.0 [3.0-4.0] (n=42)	3.0 [2.0-3.5] (n=23)	3.0 [2.0-4.0] (n=42)	3.0 [2.0-3.8] (n=38)	3.0 [2.0-4.0] (n=17)	0.012	6 months: p=0.004; 12 months: p=0.047
ATI smoke	3.0 [3.0-4.0] (n=34)	3.0 [1.2-3.8] (n=14)	3.0 [2.2-4.0] (n=34)	3.0 [2.0-3.0] (n=30)	3.0 [2.0-3.0] (n=15)	0.002	6 months: p=0.004
ATI irritants	2.0 [0.0-3.0] (n=36)	3.0 [2.0-3.0] (n=17)	1.0 [0.0-2.0] (n=36)	1.0 [0.0-2.0] (n=34)	1.0 [0.0-2.0] (n=16)	<0.001	3 months: p=0.002; 6 months: p<0.001; 12 months: p=0.008
ATI infections	3.0 [3.0-4.0] (n=41)	3.0 [2.0-4.0] (n=22)	3.0 [2.0-3.0] (n=40)	2.0 [2.0-3.0] (n=36)	2.5 [1.8-3.0] (n=16)	<0.001	3 months: p=0.012; 6 months: p<0.001; 12 months: p=0.007

Abbreviations: ATI, Asthma Trigger Inventory; IQR, interquartile range; SD, standard deviation.
